# Paucity of viral infection symptoms in patients with immune-mediated inflammatory diseases

**DOI:** 10.1136/bmjopen-2024-088486

**Published:** 2025-01-07

**Authors:** Melek Yalcin Mutlu, Arnd Kleyer, Gerhard Kroenke, Filippo Fagni, Selahattin Alp Temiz, Christine Meder, Peter Dietrich, Till Orlemann, Johanna Mößner, Verena Schoenau, Daniela Bohr, Louis Schuster, Fabian Hartmann, Ioanna Minopoulou, Moritz Leppkes, Andreas Ramming, Milena L Pachowsky, Florian Schuch, Monika Ronneberger, Stefan Kleinert, Axel J Hueber, Karin Manger, Bernhard Manger, Raja Atreya, Carola Berking, Michael Sticherling, Markus F Neurath, Georg Schett, David Simon, Koray Tascilar

**Affiliations:** 1 Universitätsklinikum Erlangen Department of Medicine-3, Rheumatology and Immunology, Friedrich-Alexander University Erlangen-Nuremberg, Erlangen, Bayern, Germany; 2 Universitätsklinikum Erlangen, Deutsches Zentrum fuer Immunotherapie, Friedrich-Alexander University Erlangen-Nuremberg, Erlangen, Bayern, Germany; 3 Department of Rheumatology and Clinical Immunology, Charite - Universitatsmedizin Berlin, Berlin, Berlin, Germany; 4 Universitätsklinikum Erlangen Department of Dermatology, Friedrich-Alexander University Erlangen-Nuremberg, Erlangen, Germany; 5 Universitätsklinikum Erlangen Department of Medicine 1, Kussmaul Research Campus & Ludwig Demling Endoscopy Center of Excellence, Friedrich-Alexander University Erlangen-Nuremberg, Erlangen, Bayern, Germany; 6 Universitätsklinikum Erlangen Department of Medicine-2, Friedrich-Alexander University Erlangen-Nuremberg, Erlangen, Bayern, Germany; 7 Rheumatology Practice Erlangen, Erlangen, Germany; 8 Division of Rheumatology, Klinikum Nürnberg, Paracelsus Private Medical University - Nuremberg Campus, Nurnberg, Bayern, Germany; 9 Rheumazentrum Erlangen-Nürnberg, UK; 10 Rheumatology Practice Bamberg, Bamberg, Bayern, Germany

**Keywords:** Respiratory infections, Rheumatology

## Abstract

**Abstract:**

**Objectives:**

Although patients with immune-mediated inflammatory diseases (IMID) are thought to be more susceptible to viral infections, it is unclear whether their presentation differs between patients with IMID and healthy controls. This study aimed to investigate the symptom pattern of common viral infections in patients with IMID and compare it with controls without IMIDs.

**Design:**

A cross-sectional study conducted between 1 February and 30 April 2020, using a questionnaire.

**Setting:**

Seven tertiary regional care centers in Germany, which specialised in the care of patients with IMID (namely, in gastroenterology, dermatology, rheumatology and immunology clinical care).

**Participants:**

One thousand nine hundred nine participants completed the survey (757 patients with IMID; 1152 non-IMID controls).

**Primary outcome measure:**

The occurrence of 11 common viral illness symptoms within the preceding 3 months in patients with IMID and non-IMID controls.

**Results:**

Symptom data were clustered, based on number and co-occurrance, into 3 major clusters and 2 subclusters ranked by the average number of symptoms. Patients with inflammatory bowel disease and psoriasis were significantly overrepresented in the lower-frequency subcluster of the polysymptomatic cluster. Patients with rheumatoid arthritis were overrepresented in the lower-frequency subclusters of the intermediate and oligo-/asymptomatic clusters. Controls were over-represented only in the higher-frequency subclusters of each major cluster where none of the IMIDs were over-represented. Spondyloarthritis and other IMIDs were also overrepresented in the low-frequency subcluster, but the results were not significant. Overall, patients with rheumatoid arthritis patients reported fewer symptoms (rate ratio=0.68, 95% CI, 0.59 to 0.80) than non-IMID controls.

**Conclusion:**

Patients with IMID are over-represented in low-frequency subclusters, even among individuals who have reported a broad range of viral infection symptoms. This pattern suggests that the manifestations of viral infections are different between patients with IMID and controls, thus challenging the accurate and early diagnosis of infections.

**STRENGTHS AND LIMITATIONS OF THIS STUDY**

- The study benefits from a large sample size of 1909 participants, including a diverse and consecutive selection of patients with IMID, which enhances the statistical power and generalisability of the findings.
- Data include a diverse sample of patients with IMID and a sizable control group from the same area and time frame.
- Symptom data were patient-reported, potentially leading to recall and reporting biases.
- Participants were not tested for intercurrent infections; therefore, a clear identification of specific viruses was not possible.

## Introduction

 Respiratory viral infections (RVIs) are among the most frequent infectious diseases and represent a significant cause of morbidity and mortality.^1^ Besides the negative effect on public health, RVIs also impose a considerable economic burden because of unnecessary antibiotic prescriptions and absence from work or school.^2^

The clinical presentation of RVIs ranges from asymptomatic viral shedding to severe life-threatening respiratory distress.^1 3 4^ The presence and intensity of symptoms are orchestrated by intricate relationships between virulence factors, host susceptibility and host response.^5^ One of the key factors in limiting viral replication is the host response.^6^ Except for influenza, human metapneumovirus, and SARS-CoV-2, more than 70% of RVIs are oligo- or asymptomatic in the general population.^7^ Upper respiratory symptoms are the most common complaint among symptomatic individuals, whereas systemic effects are less frequent.^1 8^

The majority of our knowledge of RVI symptoms originates from the general ‘healthy’ population. Evidence on the common viral infection symptoms in at-risk individuals, such as patients with immune-mediated inflammatory diseases (IMIDs), is relatively scarce. Previous studies have suggested that patients with IMID experience more frequent systemic infections than healthy controls^9^,^12^ and that this phenomenon might be influenced by not only the disease but also concomitant therapies.^13 14^ Apart from unfavourable clinical outcomes, disease-modifying anti-rheumatic drugs (DMARDs) have been linked to the increased risk of certain viral infections, such as herpes zoster reactivation and John Cunningham virus-induced progressive multifocal leucoencephalopathy, which are associated with the use of JAK inhibitors and rituximab, respectively.^15^,^17^

Although patients with IMID are considered to be at increased risk of developing systemic complications, the questions of whether RVI symptoms are different in patients with IMID compared with otherwise healthy controls and how often patients with IMID experience RVI symptoms remain unanswered. Furthermore, existing studies investigating the aetiology and epidemiology of respiratory illnesses are frequently biased toward detecting more severe infections, as a substantial proportion of study participants are usually patients who actively sought medical attention. We hypothesised that the altered immune system function in patients with IMID would also modify the expression of RVI symptoms and conducted a study to explore the breadth of common RVI symptom expression in a large cohort of patients with IMID and controls.

## Methods

### Study design and participants

The data for this cross-sectional study were obtained from the COVID-19 study programme of the Deutsches Zentrum für Immuntherapie, a prospective cohort that recruits patients with IMID and healthy controls to collect data on (1) respiratory infections, including COVID-19, (2) anti-SARS-CoV-2 antibody responses and (3) exposure risk behaviour over time since the onset of the COVID-19 pandemic. This study programme was conducted with the collaboration of regional centres specialised in the care of IMIDs (namely, in gastroenterology, dermatology, rheumatology and immunology clinical care).

A detailed explanation of cohort inclusion and data collection has previously been provided elsewhere.^18^ Patients with IMID were attendees of the participating centres, with the following diagnoses: rheumatoid arthritis (RA), inflammatory bowel disease (IBD), spondyloarthritis (SpA; axial SpA and psoriatic arthritis), psoriasis, and other IMIDs. ‘Other IMIDs’ included systemic lupus erythematosus (SLE), primary Sjogren’s syndrome, systemic sclerosis, polymyositis, IgG4-related disease, sarcoidosis, juvenile idiopathic arthritis, adult-onset Still’s disease, periodic fever syndromes, Behcet’s disease, autoimmune hepatitis, giant cell arteritis, Takayasu arteritis, granulomatosis with polyangiitis and polymyalgia rheumatica. The non-IMID participants consisted of healthcare professionals and healthy volunteers from the general population.

Patients or the public were not involved in the design, or conduct, or reporting, or dissemination plans of our research.

### Data sources

The data analysed were collected from 1 February to 30 April 2020. Using a structured questionnaire (online supplemental information file), participants were asked to indicate clinical symptoms of common viral infections experienced within the previous 3 months. Questioned symptoms were cough, nasal congestion/rhinitis (used interchangeably), throat pain/pharyngitis (used interchangeably), fever, headache, fatigue, musculoskeletal pain, loss of smell/anosmia (used interchangeably), shortness of breath and diarrhoea. The questionnaire also included demographic data, IMID diagnosis and current medications for IMID.

### Statistical analysis

Descriptive statistics were used to summarise the cohort characteristics. An unsupervised binary data clustering algorithm based on expectation maximisation^19^ was used to group individuals with similar number and co-occurrence of symptoms regardless of their diagnoses. For this, a matrix with one row per person and one column per symptom was generated, where the presence of a symptom was coded as 1 and absence as 0. A 6×4 clustering was performed, where the algorithm would identify six row clusters by grouping rows, that is, individuals, with similar numbers and types of symptoms together and four column clusters by grouping the columns based on the co-occurrence of specific symptoms, that is, qualitative clusters. The six row clusters, which were based on both the number and co-occurrence of symptoms within individuals, were ranked by the mean number of symptoms and by merging two adjacent clusters organised into three major clusters, polysymptomatic, including individuals who expressed a wide range of symptoms; oligo-/asymptomatic, indicating individuals who expressed few or none of the questioned symptoms; and intermediately symptomatic. Accordingly, each cluster included two subclusters with a higher or lower mean number of symptoms. The frequency distributions of patients with IMID and healthy controls were then explored across these clusters and subclusters. To this end, contingency tables were generated for the frequency of controls and individual IMID diagnoses in each cluster, and standardised residuals were calculated using the cell counts and marginal counts akin to a χ^2^ test. These residuals represent a standard quantity of deviation for the observed number of individuals from the expected number based on the marginal frequencies. These residuals were used as an estimate of the over-representation/under-representation of controls or individuals with IMID diagnoses in each subject cluster; a standardised residual larger than 2 was considered an important deviation.^20^ Finally, because we have counts of symptom types experienced during a fixed time in a fixed population, we used Poisson regression to compare these counts in IMID groups with those in the control group. In this model, the number of symptoms was the dependent variable, whereas age, sex and diagnosis were the independent variables. Exponentiated coefficients for diagnoses from this model represented the ratio of the number of symptoms in patients with IMID in comparison with controls after adjusting for age and sex. Analyses were conducted using the open-source R software V. 4.3.0 (R Foundation, Vienna, Austria).

## Results

### Study population and baseline characteristics

A total of 1909 study participants were included, of whom 757 (39%) were diagnosed with an IMID, and 1152 (61%) were controls (table 1). The IMID group included 226 (11%) patients with RA, 178 (9%) with IBD, 142 (7%) with SpA, 89 (4 %) with psoriasis and 122 (6 %) with other-IMIDs. The mean (SD) age of the patients with IMID and controls were 50.9 (16.1) and 41.8 (13.5) years, respectively. Male participants constituted the majority of the control group (n=744, 64.6%), whereas females predominated the IMID group (n=418, 55.2%).

**Table 1:** Baseline participant characteristics

	Overall	Controls	Overall IMIDs	RA	IBD	SpA	Other IMIDs	Psoriasis
Number of participants (n)	1909	1152	757	226	178	142	122	89
Age mean (SD)	45.4 (15.2)	41.8 (13.5)	50.9 (16.1)	59.3 (13.8)	40.6 (14.2)	49.8 (13.8)	52.2 (16.9)	50.1 (15.2)
Malen (%)	1080(56.6)	744(64.6)	336(44.4)	62(27.4)	86(48.3)	81(57.0)	52(42.6)	55(61.8)
Femalen (%)	824(43.2)	406(35.2)	418(55.2)	163(72.1)	92(51.7)	61(43.0)	69(56.6)	33(37.1)
csDMARDsn (%)	328(17.2)	–	217(28.7)	116(51.3)	7(3.9)	35(24.6)	47(38.5)	12(13.5)
bDMARDsn (%)	542(28.4)	–	438(57.9)	86(38.1)	162(91.0)	96(67.6)	42(34.4)	52(58.4)
tsDMARDsn (%)	17(0.9)	–	17(2.2)	14(6.2)	0(0.0)	1(0.7)	1(0.8)	1(1.1)
Glucocorticoidsn (%)	141(7.4)	–	141(18.6)	54(23.9)	23(12.9)	12(8.5)	48(39.3)	4(4.5)

Data are presented as mean with SD or absolute number (n) with proportion (%).

bDMARDsbiologic disease-modifying anti-rheumatic drugscsDMARDsconventional synthetic disease-modifying anti-rheumatic drugsIBDinflammatory bowel diseaseIMIDsimmune-mediated inflammatory diseasesRArheumatoid arthritisSpAspondyloarthritistsDMARDstarget synthetic disease-modifying anti-rheumatic drugs

In this study, 217 (28.7 %) of the patients with IMID received conventional synthetic disease-modifying anti-rheumatic drugs (csDMARDs), 438 (57.9 %) received biological DMARDs (bDMARDs) and 17 (2.2%) received targeted-synthetic DMARDs (tsDMARDs). Glucocorticoids were used by 141 (18.6 %) of the patients with IMID (table 1).

### Frequency and types of symptoms in patients with IMID and controls

The most commonly reported symptoms in the overall cohort were rhinitis (618 participants, 32.4%), pharyngitis (422 participants, 22.1%) and headache (476 participants, 24.9%) (table 2). Shortness of breath, fever and anosmia were the least frequent complaints (less than 10%). The overall type and frequency of viral symptoms in the control group were similar to the overall cohort, the most frequent being rhinitis with a frequency of 38.2% (n=440) and the least frequent being anosmia with a frequency of 2.3% (n=26). Likewise, rhinitis and headache were the most common symptoms in all IMID groups ranging from 17.7% to 30.9% (table 2). Comparable with the controls, anosmia was the least common symptom (19 participants, 2.5%) among patients with IMID.

**Table 2:** Mean number of symptoms and frequencies according to diagnosis

	Overall	Controls	Overall IMIDs	RA	IBD	SpA	Other IMIDs	Psoriasis
Number of symptoms mean (SD)	1.2 (1.7)	1.3 (1.7)	1.2 (1.6)	1.0 (1.5)	1.5 (1.7)	1.1 (1.7)	1.4 (1.7)	1.2 (1.7)
Rhinitisn (%)	618 (32.4)	440 (38.2)	178 (23.5)	41 (18.1)	55 (30.9)	34 (23.9)	23 (18.9)	25 (28.1)
Headachen (%)	476 (24.9)	313 (27.2)	163 (21.5)	40 (17.7)	49 (27.5)	26 (18.3)	26 (21.3)	22 (24.7)
Pharyngitisn (%)	422 (22.1)	305 (26.5)	117 (15.5)	22 (9.7)	33 (18.5)	23 (16.2)	27 (22.1)	12 (13.5)
Coughn (%)	330 (17.3)	223 (19.4)	107 (14.1)	30 (13.3)	22 (12.4)	16 (11.3)	21 (17.2)	18 (20.2)
Fatiguen (%)	285 (14.9)	162 (14.1)	123 (16.2)	26 (11.5)	44 (24.7)	23 (16.2)	21 (17.2)	9 (10.1)
Diarrhoean (%)	216 (11.3)	106 (9.2)	110 (14.5)	24 (10.6)	54 (30.3)	11 (7.7)	9 (7.4)	12 (13.5)
Musculoskeletal painn (%)	175 (9.2)	87 (7.6)	88 (11.6)	37 (16.4)	13 (7.3)	18 (12.7)	12 (9.8)	8 (9.0)
Night sweatsn (%)	173 (9.1)	90 (7.8)	83 (11.0)	20 (8.8)	15 (8.4)	17 (12.0)	21 (17.2)	10 (11.2)
Shortness of breathn (%)	131 (6.9)	68 (5.9)	63 (8.3)	14 (6.2)	18 (10.1)	11 (7.7)	11 (9.0)	9 (10.1)
Fevern (%)	114 (6.0)	73 (6.3)	41 (5.4)	6 (2.7)	10 (5.6)	8 (5.6)	12 (9.8)	5 (5.6)
Anosmian (%)	45 (2.4)	26 (2.3)	19 (2.5)	4 (1.8)	3 (1.7)	3 (2.1)	6 (4.9)	3 (3.4)

Data are presented as mean with SD or absolute number (n) with proportion (%).

IBDinflammatory bowel diseaseIMIDsimmune-mediated inflammatory diseasesRArheumatoid arthritisSDstandard deviationSpAspondyloarthritis

The mean number (SD) of symptoms per patient in the IMID group was lower (1.2 (1.6)) than that in the control group (1.3 (1.7)). Patients with RA showed the lowest number of symptoms among all IMID groups, with a mean symptom count of 1.0 (1.3), followed by SpA (1.1±1.7) and psoriasis (1.2±1.7). Compared with the control group, the mean number of symptoms was higher only in the IBD (1.5±1.7) and ‘other IMIDs’ (1.4±1.7)groups.

### Clusters of common viral infection symptoms according to frequency and co-occurrence

**Figure 1:**
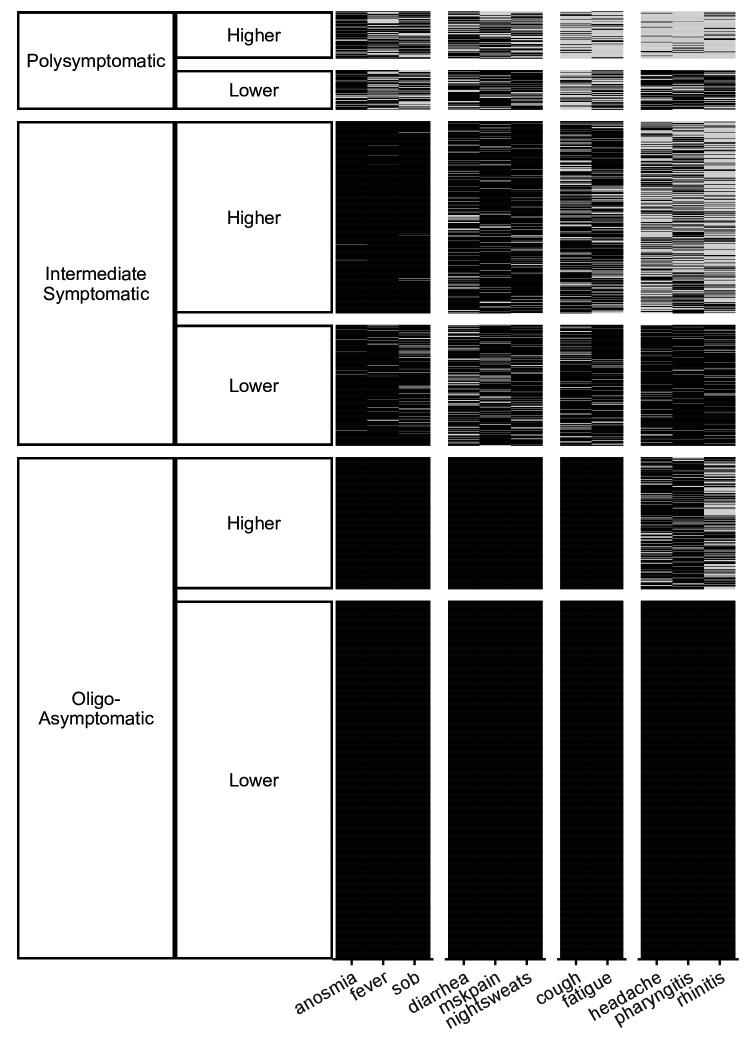
Cluster plot: Clusters according to symptom type and number. The most frequent symptom group was comprised of headache, pharyngitis and rhinitis, whereas the least frequent symptom group constituted shortness of breath, anosmia and fever (X-axis). Three major clusters, namely, polysymptomatic, intermediately symptomatic and oligo-/asymptomatic, were formed. Each cluster was divided into two subclusters showing higher and lower symptom frequencies (Y-axis).

The most frequent symptoms were headache, pharyngitis, and rhinitis, whereas the least frequent were shortness of breath, anosmia and fever (figure 1, X-axis). Symptoms with intermediate frequency were grouped into cough and fatigue or diarrhoea, musculoskeletal pain and night sweats.

According to the number of symptoms, three main clusters were generated: (1) polysymptomatic, indicating individuals who, within the previous 3 months, had experienced a broad range of the 11 symptoms that were questioned; (2) intermediate symptomatic, indicating limited range of reported symptoms; and (3) oligo/asymptomatic, indicating individuals who had experienced only a minority or none of the 11 symptoms within the previous 3 months. Additionally, each of these main clusters was divided into lower and higher frequency subclusters (figure 1, Y-axis). The mean (SD) number of symptoms in the higher and lower frequency subclusters of the polysymptomatic cluster were 6.0±1.3 and 3.9±1.1, respectively. Respective numbers were 2.2±1.0 and 1.5±0.6 for the intermediate symptomatic cluster and 0.5±0.5 and 0 in the oligo/asymptomatic cluster.

### Assessment of the deviation from expected group frequencies across clusters

**Figure 2:**
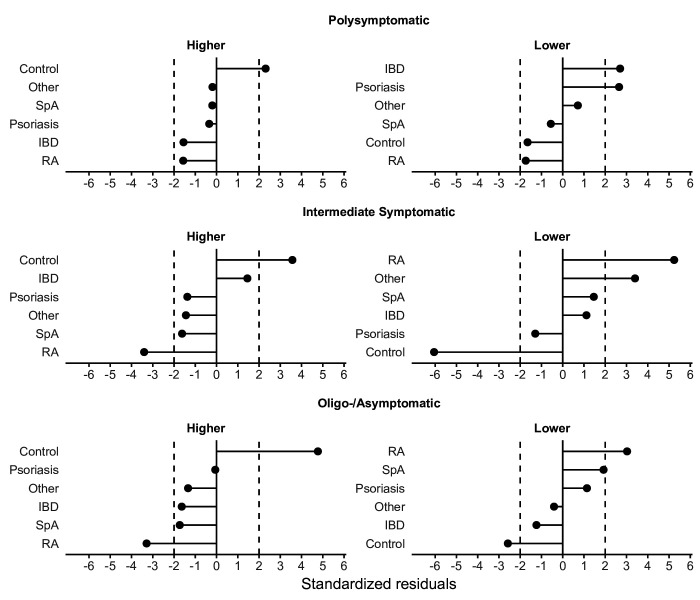
Standardised residuals that summarise the difference between the observed and expected number of individuals in each cluster. Values <−2 or >2 were interpreted as an important deviation from the expected counts. Standardised residuals by diagnosis: Controls showed the largest positive deviations from the expected number of participants and over-represented in the high subcluster of each symptom frequency group. Conversely, patients with IMID predominated the low-symptom subclusters in each cluster. RA displayed the largest deviations. IBD, inflammatory bowel disease; IMID, immune-mediated inflammatory disease; RA, rheumatoid arthritis; SpA, spondyloarthritis.

To explore how the frequencies of individual IMID diagnoses and controls diverge across the clusters, the standardised residuals for the IMID and control groups were calculated in each frequency cluster (figure 2, online supplemental tables 1 and 2). A detailed summary of the observed and expected number of participants and standardised residuals are presented in online supplemental table 2. Controls showed the largest positive deviations from the expected number of participants in the high-frequency subclusters across all clusters, suggesting that in a similar symptom–frequency range represented by the clusters, controls were more likely to express a wider range of symptoms than patients with IMID. Patients with RA showed the largest negative deviations, indicating that they were enriched in the low-frequency subclusters. For patients with RA, enrichment in the low-frequency subclusters was observed in all three clusters. In patients with SpA, this enrichment was only observed in the intermediate and oligo-asymptomatic clusters. Analysing systemic autoimmune diseases, that is, SLE, autoimmune inflammatory myopathies and systemic sclerosis in a common category, did not affect our conclusions (online supplemental figure 1).

The associations between frequency clusters and types of treatment in patients with IMID were also analysed by estimating standardised residuals for csDMARDs, bDMARDs, tsDMARDs and glucocorticoids (onlinesupplemental figure 2A  2B, table 3) and including specific targeted treatments (online supplemental figure 3). The deviations from the expected numbers by clusters followed a pattern similar to the analysis for diagnosis. Regardless of the granularity of the stratification, we were unable to discern a particular pattern for the deviations from the expected counts according to treatment.

### Number of reported viral infection symptoms in patients with IMID versus controls

To explore the influence of individual IMID diagnoses on the number of reported symptoms, a Poisson regression analysis was performed (table 3). This analysis indicates that RA was associated with a 32% reduction in the number of symptoms compared with controls (rate ratio, 0.68, 95% CI, 0.59 to 0.80) after adjusting for age and sex. Although the mean number of symptoms was also different between controls and patients with IBD and patients with other IMIDs, significance disappeared after correcting for age and sex.

**Table 3:** Poisson regression analysis for the number of viral symptoms

	Rate ratio (95% CI)	P value
Age (per decade)	0.96 (0.94 to 0.99)	0.014
Female sex	1.72 (1.59 to 1.87)	<0.001
Control	1 (Ref.)	–
RA	0.68 (0.59 to 0.80)	<0.001
IBD	1.06 (0.92 to 1.20)	0.425
SpA	0.86 (0.72 to 1.01)	0.072
Other IMIDs	0.99 (0.84 to 1.16)	0.897
Psoriasis	0.99 (0.81 to 1.20)	0.895

IBDinflammatory bowel diseaseIMIDsimmune-mediated inflammatory diseasesRArheumatoid arthritisSpAspondyloarthritis

This analysis also indicated that increasing age was associated with lower symptom counts (rate ratio per decade, 0.96, 95% CI, 0.94 to 0.99), whereas female sex was associated with higher symptom counts (rate ratio, 1.72; 95%, 1.59 to 1.87).

## Discussion

This study shows that patients with IMID exhibit a narrower range of symptoms associated with viral infection than healthy controls. To our knowledge, this is the first study exploring the frequency and clusters of viral infection symptoms in the IMID population. Certain symptoms appear to co-occur with varying frequencies. Headache, rhinitis and pharyngitis were the components of the most frequent cluster with the highest prevalence, whereas anosmia, shortness of breath and fever were represented in the least common cluster. Previous studies have reported resembling clinical expression patterns 21,23; nonetheless, our results go beyond prior research in terms of clustering as well as a detailed presentation of the number and type of viral symptoms.

Few studies have assessed the frequency of viral symptoms in patients with IMID, mainly in the COVID-19 era.^21^,^23^ Comparable to our analysis, the paucity of viral symptoms was demonstrated in a cohort of more than 2000 patients with IMID. Regardless of the treatment modality, the symptom prevalence was lower in patients with IMID with serologically confirmed infection than in the general population.^22 23^ The most frequently detected symptoms were upper respiratory tract symptoms, fatigue and headache. Akin to these findings, rhinitis and pharyngitis, together with headache, formed the most common symptom cluster in this study. The BELCOMID study showed high-grade fever and anosmia as the second most common complaints.^22 23^ Contrarily, shortness of breath, anosmia and fever constituted the least common symptom cluster. Besides, the frequency of each symptom was significantly higher in the aforementioned studies than in the present study.^21^,^23^ These discrepancies likely result from the fact that SARS-CoV-2 infection, addressed in the BELCOMID study, has a different clinical course from other common human respiratory viruses, such as rhinovirus, adenovirus and respiratory syncytial virus,^1^ particularly before the appearance of milder SARS-CoV-2 variants.

In this study, we did not find a noteworthy pattern between the type of immunomodulatory treatment and the frequency of symptoms. In contrast, another pre-print research in a large cohort noted that patients who were exposed to DMARDs, whether csDMARDs or b/ts DMARDs, showed fewer symptoms than those who were not.^24^ However, a considerable proportion of this cohort consisted of osteoarthritis patients. Hence, we cannot conclude whether the treatment-associated symptom paucity was due to the lack of treatment exposure per se or the distinct host characteristics of osteoarthritis patients in contrast to patients with IMID. So far, current data do not allow us to delineate the effect of treatment on symptom occurrence.

The clinical symptoms of RVIs are influenced partially by the type of virus but to a greater extent by host characteristics, such as age, sex and the physiological and immunological state of the host.^6^ Age and sex associations were detected across the overall population regardless of the IMID status, and this is a well-known phenomenon, that is, viral infection symptoms are less likely to be manifested with increasing age and in males than in females^1 25^ Our aim in including age and sex in the analyses was not necessarily for reproduction or contention of this pattern but was principally for adjustment because patients with IMID were older and were more likely to be females.

Interestingly, recent studies have suggested that viral infections tend to follow an asymptomatic course in individuals who develop an early strong inflammatory response to viral exposure.^26^ Interferons (IFNs) play a crucial role in this early phase of infections, as a consequence of their crucial role in promoting viral clearance, tissue repair and inducing adaptive immune responses.^27^,^31^ The employment of IFN-based therapies against viral infections, such as hepatitis C virus and COVID-19, further substantiates the potent antiviral effect.^32 33^ Moreover, this concept is paralleled by lower disease activity but a higher risk of herpes zoster in patients with SLE with pre-existing autoantibodies neutralising IFN-α.^34^

Notably, IFNs have attracted attention in IMIDs in light of studies showing pronounced type 1 IFN signatures in SLE, RA, systemic sclerosis, Sjögren’s syndrome and psoriasis.^35^,^39^ Due to the robust antiviral effect of IFNs during immune homeostasis, its increased expression in patients with IMID might hint at a connection between the scarcity of viral symptoms and the presence of sustained IFN responses in patients with IMID. Furthermore, IFNs mediate symptoms such as fatigue and headache, which are prevalent in patients with IMID.^5 21 22 40^ Therefore, this altered expression of and/or response to IFNs in patients with IMID could be a plausible explanation for the altered expression of viral infection symptoms. Conversely, whether an inappropriately sustained or exaggerated IFN response as observed in various IMIDs is effective in viral clearance as it is during immune homeostasis is not clearly established, and how this sustained ‘IFN signature’ per se relates to the expression of common RVI symptoms is also unclear. In fact, IFN production against various stimuli and from various sources has been well studied, and the overall increasing production of IFN gamma by age and in females supports the observed patterns of viral symptom expression with age and sex. However, the early IFN response to viral encounters with plasmacytoid dendritic cells is important for viral clearance, and the response of these cells to viral stimuli seems to be dampened with age,^41^ which is also the case in autoimmunity.^42^ Therefore, the timely initiation and the appropriate regulation of the intensity of the overall immune response to a viral infection probably determine the balance between the effectiveness of viral clearance and symptom expression.^43^ A disruption in this balance could be a result of dysregulated inflammation, which leads to an inflammatory disease while causing an altered immune response to viral infections altering symptom expression as we have observed.

This study has some limitations. Symptom data were patient-reported, and given the survey study design, it was prone to recall bias. However, compared with controls, patients with IMID could also be more likely to inform healthcare professionals about their symptoms, which might lead to over-reporting. Second, participants were not tested for intercurrent infections; therefore, a clear identification of specific viruses was not possible. Third, the clustering method used in this study does not account for age and sex differences between groups, which may have confounded the associations observed; however, results of the adjusted analysis also support the notion that patients with IMID, particularly those with RA, express a narrower range of RVI symptoms in comparison with controls. The large sample size and the inclusion of different consecutive/unselected patients with IMID are the major strengths of this study. Another strength is the availability of symptom data from a sizeable control group residing in the same area collected in the same time frame instead of relying on previous studies in the general population.

In conclusion, symptoms of common viral respiratory infections are restrained in patients with IMID, especially in RA. These results suggest that the underlying chronic inflammatory disease and/or anti-inflammatory treatment modulate the expression of common RVI symptoms.

## supplementary material

10.1136/bmjopen-2024-088486 online supplemental file 1

10.1136/bmjopen-2024-088486 online supplemental file 2

10.1136/bmjopen-2024-088486 online supplemental file 3

10.1136/bmjopen-2024-088486 online supplemental file 4

10.1136/bmjopen-2024-088486 online supplemental file 5

10.1136/bmjopen-2024-088486 online supplemental file 6

10.1136/bmjopen-2024-088486 online supplemental file 7

## Data Availability

Data are available upon reasonable request.

## References

[1] Byington CL, Ampofo K, Stockmann C (2015). Community Surveillance of Respiratory Viruses Among Families in the Utah Better Identification of Germs-Longitudinal Viral Epidemiology (BIG-LoVE) Study. Clin Infect Dis.

[2] Legand A, Briand S, Shindo N (2013). Addressing the Public Health Burden of Respiratory Viruses: The Battle Against Respiratory Viruses (BRaVe) Initiative. Future Virol.

[3] Monto AS (1995). Viral respiratory infections in the community: epidemiology, agents, and interventions. Am J Med.

[4] Jansen RR, Wieringa J, Koekkoek SM (2011). Frequent detection of respiratory viruses without symptoms: toward defining clinically relevant cutoff values. J Clin Microbiol.

[5] Eccles R (2005). Understanding the symptoms of the common cold and influenza. Lancet Infect Dis.

[6] Troy NM, Bosco A (2016). Respiratory viral infections and host responses; insights from genomics. Respir Res.

[7] Galanti M, Birger R, Ud-Dean M (2019). Rates of asymptomatic respiratory virus infection across age groups. Epidemiol Infect.

[8] Kim GU, Kim MJ, Ra SH (2020). Clinical characteristics of asymptomatic and symptomatic patients with mild COVID-19. Clin Microbiol Infect.

[9] Blumentals WA, Arreglado A, Napalkov P (2012). Rheumatoid arthritis and the incidence of influenza and influenza-related complications: a retrospective cohort study. BMC Musculoskelet Disord.

[10] D’Silva KM, Serling-Boyd N, Wallwork R (2020). Clinical characteristics and outcomes of patients with coronavirus disease 2019 (COVID-19) and rheumatic disease: a comparative cohort study from a US 'hot spot'. Ann Rheum Dis.

[11] Fragoulis GE, Bournia V-K, Sfikakis PP (2022). Different systemic rheumatic diseases as risk factors for COVID-19-related mortality. Clin Rheumatol.

[12] Pablos JL, Galindo M, Carmona L (2020). Clinical outcomes of hospitalised patients with COVID-19 and chronic inflammatory and autoimmune rheumatic diseases: a multicentric matched cohort study. Ann Rheum Dis.

[13] Greenberg SB (2002). Infections in the immunocompromised rheumatologic patient. Crit Care Clin.

[14] Winthrop KL, Novosad SA, Baddley JW (2015). Opportunistic infections and biologic therapies in immune-mediated inflammatory diseases: consensus recommendations for infection reporting during clinical trials and postmarketing surveillance. Ann Rheum Dis.

[15] Bechman K, Subesinghe S, Norton S (2019). A systematic review and meta-analysis of infection risk with small molecule JAK inhibitors in rheumatoid arthritis. Rheumatology (Oxford).

[16] Olivera PA, Lasa JS, Bonovas S (2020). Safety of Janus kinase inhibitors in patients with inflammatory bowel diseases or other immune-mediated diseases: a systematic review and meta-analysis. Gastroenterology.

[17] Bennett CL, Focosi D, Socal MP (2021). Progressive multifocal leukoencephalopathy in patients treated with rituximab: a 20-year review from the Southern Network on Adverse Reactions. Lancet Haematol.

[18] Simon D, Tascilar K, Krönke G (2020). Patients with immune-mediated inflammatory diseases receiving cytokine inhibitors have low prevalence of SARS-CoV-2 seroconversion. Nat Commun.

[19] Bhatia PS, Iovleff S, blockcluster GG (2017). An R Package for Model-Based Co-Clustering. J Stat Softw.

[20] Agresti A (2007). An introduction to categorical data analysis: Wiley series in probability and statistics.

[21] Bakasis A-D, Mavragani CP, Boki KA (2021). COVID-19 infection among autoimmune rheumatic disease patients: Data from an observational study and literature review. J Autoimmun.

[22] Hillary T, Van Laethem A, Castelijns F (2021). 067 BELCOMID: BELgian Cohort study of COVID-19 in Immune Mediated Inflammatory Diseases. J Invest Dermatol.

[23] Geldof J, Truyens M, Sabino J (2021). P440 First results of the BELCOMID study: BELgian Cohort study of COVID-19 in Immune Mediated Inflammatory Diseases (IMID). J Crohn's Colitis.

[24] Domenech NS, Tio L, Onaindia JL (2020). Pre-exposure to anti-tnfα decreases covid-19 symptoms: a multicentre retrospective cohort study (pre print). Authorea.

[25] Birger R, Morita H, Comito D (2018). Asymptomatic Shedding of Respiratory Virus among an Ambulatory Population across Seasons. mSphere.

[26] Xie C, Li Q, Li L (2021). Association of Early Inflammation with Age and Asymptomatic Disease in COVID-19. J Inflamm Res.

[27] Lopez J, Mommert M, Mouton W (2021). Early nasal type I IFN immunity against SARS-CoV-2 is compromised in patients with autoantibodies against type I IFNs. J Exp Med.

[28] Zhang Q, Bastard P, Liu Z (2020). Inborn errors of type I IFN immunity in patients with life-threatening COVID-19. Science.

[29] Chandran A, Rosenheim J, Nageswaran G (2022). Rapid synchronous type 1 IFN and virus-specific T cell responses characterize first wave non-severe SARS-CoV-2 infections. *Cell Rep Med*.

[30] Barrat FJ, Crow MK, Ivashkiv LB (2019). Interferon target-gene expression and epigenomic signatures in health and disease. Nat Immunol.

[31] Watson A, Spalluto CM, McCrae C (2020). Dynamics of IFN-β Responses during Respiratory Viral Infection. Insights for Therapeutic Strategies. Am J Respir Crit Care Med.

[32] Bhattacharjee C, Singh M, Das D (2021). Current therapeutics against HCV. Virusdisease.

[33] Mantlo E, Bukreyeva N, Maruyama J (2020). Antiviral activities of type I interferons to SARS-CoV-2 infection. Antiviral Res.

[34] Mathian A, Breillat P, Dorgham K (2022). Lower disease activity but higher risk of severe COVID-19 and herpes zoster in patients with systemic lupus erythematosus with pre-existing autoantibodies neutralising IFN-α. Ann Rheum Dis.

[35] Cooles FAH, Anderson AE, Lendrem DW (2018). The interferon gene signature is increased in patients with early treatment-naive rheumatoid arthritis and predicts a poorer response to initial therapy. J Allergy Clin Immunol.

[36] Brkic Z, van Bon L, Cossu M (2016). The interferon type I signature is present in systemic sclerosis before overt fibrosis and might contribute to its pathogenesis through high BAFF gene expression and high collagen synthesis. Ann Rheum Dis.

[37] Mai L, Asaduzzaman A, Noamani B (2021). The baseline interferon signature predicts disease severity over the subsequent 5 years in systemic lupus erythematosus. Arthritis Res Ther.

[38] Marketos N, Cinoku I, Rapti A (2019). Type I interferon signature in Sjögren’s syndrome: pathophysiological and clinical implications. Clin Exp Rheumatol.

[39] Zhang L (2019). Type1 Interferons Potential Initiating Factors Linking Skin Wounds With Psoriasis Pathogenesis. Front Immunol.

[40] Smith RS (1992). The cytokine theory of headache. Med Hypotheses.

[41] Zacca ER, Crespo MI, Acland RP (2015). Aging Impairs the Ability of Conventional Dendritic Cells to Cross-Prime CD8+ T Cells upon Stimulation with a TLR7 Ligand. PLoS One.

[42] Psarras A, Alase A, Antanaviciute A (2020). Functionally impaired plasmacytoid dendritic cells and non-haematopoietic sources of type I interferon characterize human autoimmunity. Nat Commun.

[43] Feng E, Balint E, Poznanski SM (2021). Aging and Interferons: Impacts on Inflammation and Viral Disease Outcomes. Cells.

