# Supplementary material for: Paucity of viral infection symptoms in patients with immune-mediated inflammatory diseases
Source: BMJ Open. 2025 Jan 7;15(1):e088486. doi: 10.1136/bmjopen-2024-088486 (PMC11749532; doi:10.1136/bmjopen-2024-088486)
Supplement: online supplemental file 1 [file bmjopen-15-1-s001.docx]

Nachname / *Last name*: ____________________________

**LAURIS-Etikett**

mit **Patient Nr.**

Vorname / *First name*: ____________________________

Geburtsdatum / *Date of Birth*: ___________________________

**Symptomatik / *Symptoms*:**

Hatten Sie **eines** oder **mehrere** der nachfolgend aufgeführten Symptome im vergangenen Zeitraum seit dem **01. Februar 2020**?

*Have you had one or more of the following symptoms in the last period since* ***February 1, 2020****?*

| 🞏 Fieber / *Fever* | 🞏 Reizhusten / *Cough* |
| --- | --- |
| 🞏 Kurzatmigkeit / *Shortness of Breath*  *or difficulty breathing* | 🞏 Erschöpfung / *Tiredness* |
| 🞏 Verstopfte Nase / *Nasal Congestion* | 🞏 Laufende Nase / *Runny Nose* |
| 🞏 Halsschmerzen / *Sore Throat* | 🞏 Durchfall / *Diarrhea* |
| 🞏 Schmerzen / *Aches* | 🞏 Geruchsverlust (Anosmie) /  *Loss of smell (Anosmia)* |
