# Supplementary material for: Paucity of viral infection symptoms in patients with immune-mediated inflammatory diseases
Source: BMJ Open. 2025 Jan 7;15(1):e088486. doi: 10.1136/bmjopen-2024-088486 (PMC11749532; doi:10.1136/bmjopen-2024-088486)
Supplement: online supplemental file 2 [file bmjopen-15-1-s002.pdf]

M3 SARS-CoV-2	Questionnaire	Universitätsklinikum Erlangen
Subject ID: __ __ __ __	Patient<br>Date: _ _ / _ _ _ / _ _ _ _	

LAURIS-Etikett

mit <u>Patient Nr.</u>

Nachname: ____________________ Vorname: ______________________

Geburtsdatum: _________________

Größe (cm): ________________ Gewicht (kg): ________________

Raucher*:* ☐ Ja ☐ Früher ☐ Nein

Name Immunerkankung (z.B. RA): ___________________________

Art Immuntherapie (z.B. adalimumab): ___________________________

Glukokortikoide: ☐ Ja ☐ Nein

Leiden Sie **<u>derzeit</u>** an **einer** oder an **mehreren** der hier aufgeführten Erkrankungen?

☐ Diabetes ☐ Bluthochdruck ☐ Thrombose ☐ Asthma

☐ Pollenallergie ☐ Rheuma ☐ Darmentzündung ☐ Schuppenflechte

☐ Neurodermitis

Wurden bei Ihnen bereits **<u>in der Vergangenheit</u> eine** oder **mehrere** der nachfolgend aufgeführten Erkrankungen festgestellt?

☐ Herzinfarkt ☐ Angina pectoris ☐ Krebserkrankung

Risiko:

Hatten Sie in **<u>den letzten 8 Wochen</u>** Kontakt mit Personen, die einen fieberhaften Atemwegsinfekt aufwiesen?

☐ Ja ☐ Nein

Waren Sie in **<u>den letzten 8 Wochen</u>** in einem Risikogebiet bzw. hatten Sie direkten Kontakt zu einem bestätigten Verdachtsfall mit SARS-CoV-2?

☐ Ja ☐ Nein

Wurden Sie bereits mittels Rachenabstrich auf eine potentielle SARS-CoV-2-Infektion getestet?

☐ Nein ☐ Ja **mit dem Testergebnis** ☐ positiv ☐ negativ

Haben Sie sich in **<u>den letzten 8 Wochen</u>** an die Vorgaben zur räumlichen Distanzierung (social distancing) gehalten?

☐ Ja ☐ Nein

Waren Sie in **<u>den letzten 8 Wochen</u>** beruflich im Home-office tätig?

☐ Ja ☐ Nein ☐ im Ruhestand/arbeitssuchend

Symptomatik:

Hatten Sie **eines** oder **mehrere** der nachfolgend aufgeführten Symptome in den vergangenen **<u>8 Wochen</u>**?

☐ Fieber
☐ Reizhusten
☐ Schnupfen
☐ Halsschmerzen
☐ Kopfschmerz
☐ Kurzatmigkeit
☐ Durchfall
☐ Gliederschmerzen
☐ Neu-aufgetretene Erschöpfung
☐ Geruchsverlust
☐ Nachtschweiß
