# Supplementary figures and images for: Paucity of viral infection symptoms in patients with immune-mediated inflammatory diseases
Source: BMJ Open. 2025 Jan 7;15(1):e088486. doi: 10.1136/bmjopen-2024-088486 (PMC11749532; doi:10.1136/bmjopen-2024-088486)

## Polysymptomatic

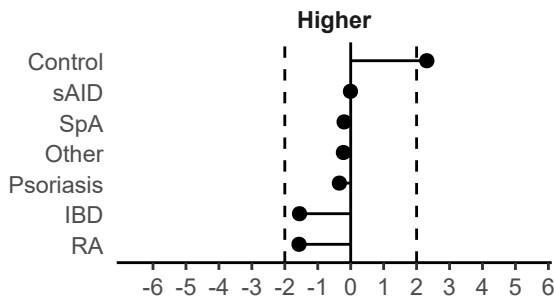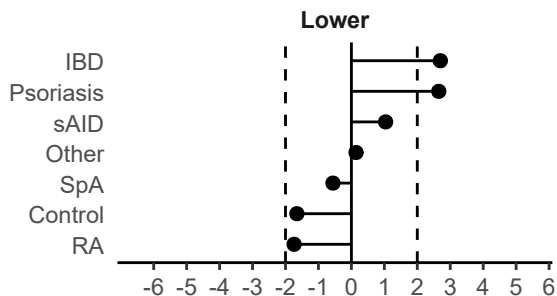

## Intermediate Symptomatic

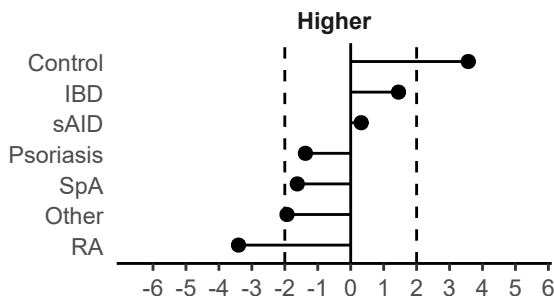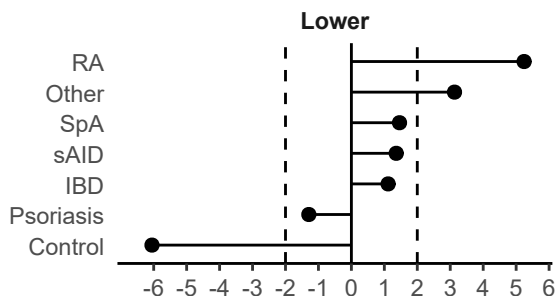

## Oligo-/Asymptomatic

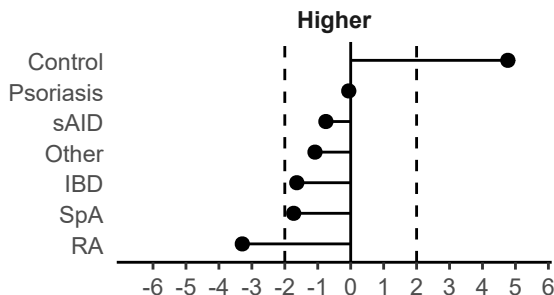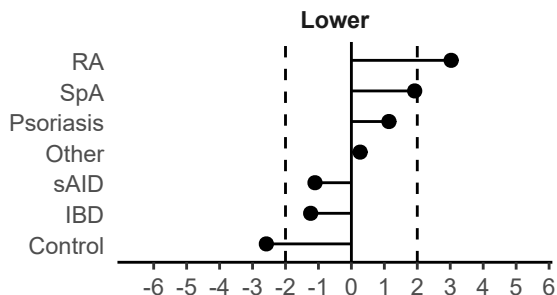

Standardized residuals

Supplement: online supplemental file 3 [file bmjopen-15-1-s003.pdf]

## Polysymptomatic

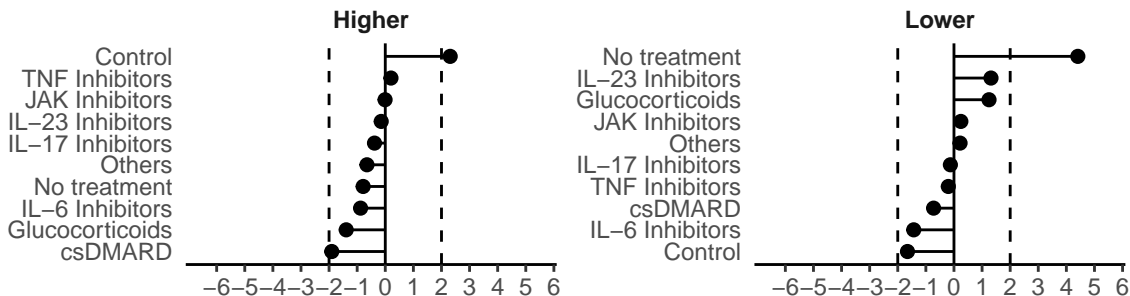

## Intermediate Symptomatic

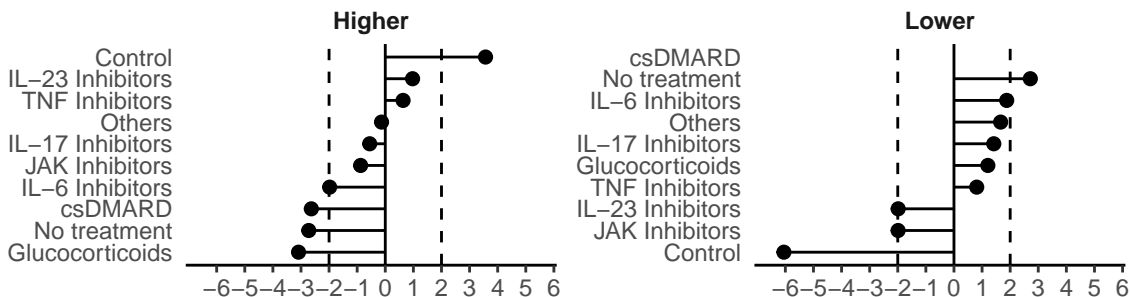

## Oligo-/Asymptomatic

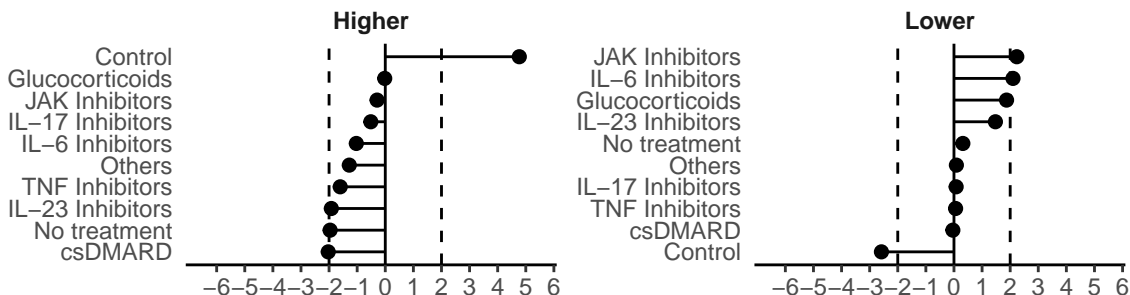

Standardized residuals

Supplement: online supplemental file 5 [file bmjopen-15-1-s005.pdf]
