# Supplementary material for: Paucity of viral infection symptoms in patients with immune-mediated inflammatory diseases
Source: BMJ Open. 2025 Jan 7;15(1):e088486. doi: 10.1136/bmjopen-2024-088486 (PMC11749532; doi:10.1136/bmjopen-2024-088486)
Supplement: online supplemental file 4 [file bmjopen-15-1-s004.pdf]

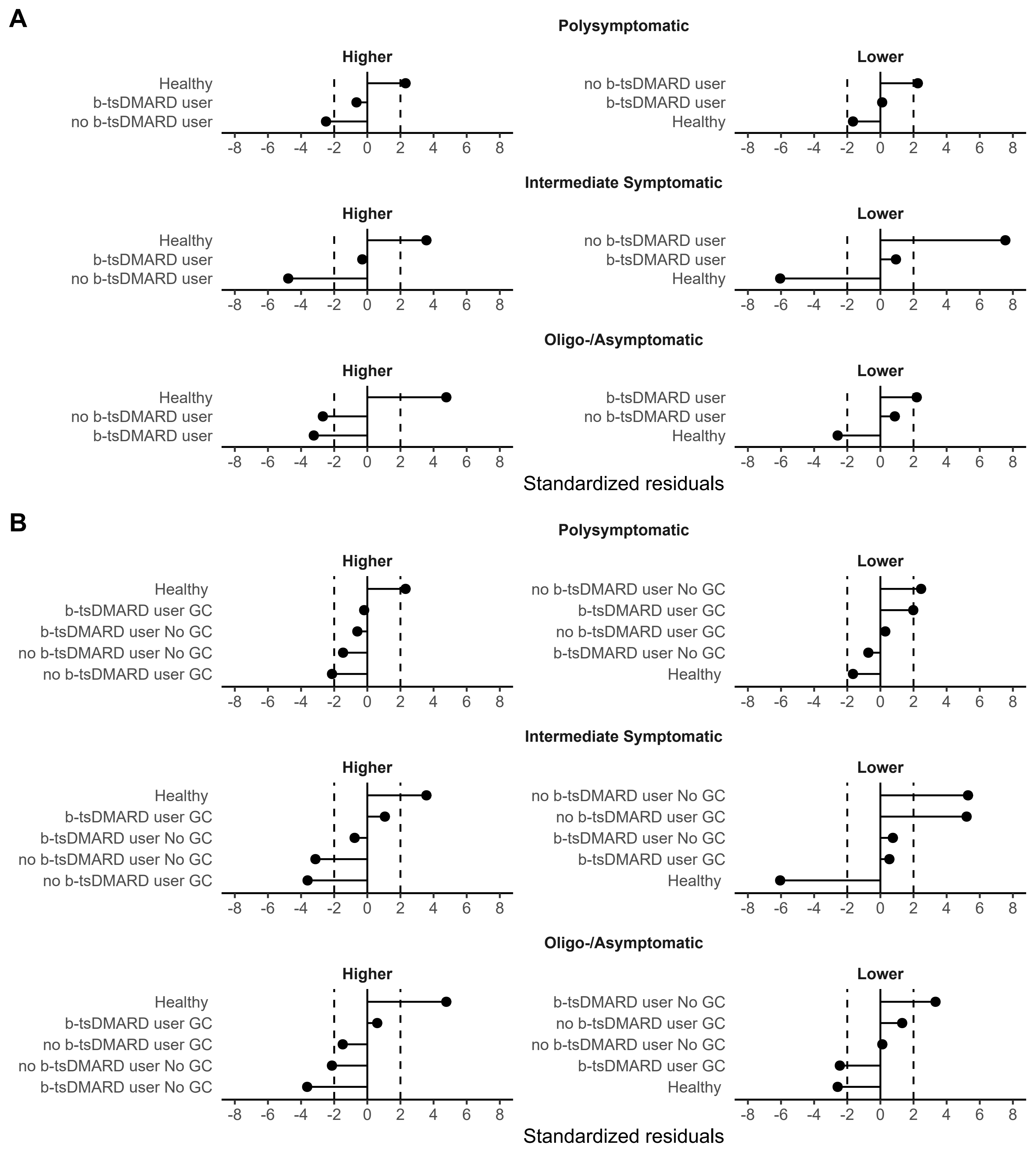
A
Polysymptomatic
Higher
Healthy
b-tsDMARD user
no b-tsDMARD user
Lower
no b-tsDMARD user
b-tsDMARD user
Healthy
Intermediate Symptomatic
Higher
Healthy
b-tsDMARD user
no b-tsDMARD user
Lower
no b-tsDMARD user
b-tsDMARD user
Healthy
Oligo-/Asymptomatic
Higher
Healthy
no b-tsDMARD user
b-tsDMARD user
Lower
b-tsDMARD user
no b-tsDMARD user
Healthy
-8 -6 -4 -2 0 2 4 6 8
Standardized residuals
B
Polysymptomatic
Higher
Healthy
b-tsDMARD user GC
b-tsDMARD user No GC
no b-tsDMARD user No GC
no b-tsDMARD user GC
Lower
no b-tsDMARD user No GC
b-tsDMARD user GC
no b-tsDMARD user GC
b-tsDMARD user No GC
Healthy
Intermediate Symptomatic
Higher
Healthy
b-tsDMARD user GC
b-tsDMARD user No GC
no b-tsDMARD user No GC
no b-tsDMARD user GC
Lower
no b-tsDMARD user No GC
no b-tsDMARD user GC
b-tsDMARD user No GC
b-tsDMARD user GC
Healthy
Oligo-/Asymptomatic
Higher
Healthy
b-tsDMARD user GC
no b-tsDMARD user GC
no b-tsDMARD user No GC
b-tsDMARD user No GC
Lower
b-tsDMARD user No GC
no b-tsDMARD user GC
no b-tsDMARD user No GC
b-tsDMARD user GC
Healthy
-8 -6 -4 -2 0 2 4 6 8
Standardized residuals
