# Supplementary material for: Paucity of viral infection symptoms in patients with immune-mediated inflammatory diseases
Source: BMJ Open. 2025 Jan 7;15(1):e088486. doi: 10.1136/bmjopen-2024-088486 (PMC11749532; doi:10.1136/bmjopen-2024-088486)
Supplement: online supplemental file 6 [file bmjopen-15-1-s006.docx]

| **Supp. Table 1: The distribution of participants by frequency clusters** | | | | | | | | | | |
| --- | --- | --- | --- | --- | --- | --- | --- | --- | --- | --- |
| Symptom frequency clusters | Subclusters of symptom frequency | | | Overall | Control | RA | IBD | SpA | Other-IMIDs | Psoriasis |
| Poly- symptomatic | | Higher  n (%) | 101 (5.3) | | 72 (6.2) | 7 (3.1) | 5 (2.8) | 7 (4.9) | 6 (4.9) | 4 (4.5) |
|  |  | Lower  n (%) | 85 (4.5) | | 44 (3.8) | 5 (2.2) | 15 (8.4) | 5 (3.5) | 7 (5.7) | 9 (10.1) |
| Intermediate Symptomatic | | Higher  n (%) | 412 (21.6) | | 280 (24.3) | 29 (12.8) | 46 (25.8) | 23 (16.2) | 20 (16.4) | 14 (15.7) |
|  |  | Lower  n (%) | 259 (13.6) | | 112 (9.7) | 56 (24.8) | 29 (16.3) | 25 (17.6) | 29 (23.8) | 8 (9.0) |
| Oligo-/ Asymptomatic | | Higher  n (%) | 283 (14.8) | | 207 (18.0) | 17 (7.5) | 19 (10.7) | 14 (9.9) | 13 (10.7) | 13 (14.6) |
|  |  | Lower  n (%) | 769 (40.3) | | 437 (37.9) | 112 (49.6) | 64 (36.0) | 68 (47.9) | 47 (38.5) | 41 (46.1) |
| Data are presented as absolute number (n) with proportion (%). IBD: inflammatory bowel disease, IMIDs: immune mediated inflammatory diseases, RA: rheumatoid arthritis, SpA: spondyloarthritis. | | | | | | | | | | |

| **Supp. Table 2: Standardized residuals by diagnosis** | | | | | | | |
| --- | --- | --- | --- | --- | --- | --- | --- |
|  | | Higher subcluster | | | Lower subcluster | | |
| Main cluster | Diagnosis | observed (n) | expected (n) | Std. Residual | observed (n) | expected (n) | Std. Residual |
| Polysymptomatic | Control | 72 | 60.95 | 2.31 | 44 | 51.29 | -1.65 |
|  | RA | 7 | 11.96 | -1.57 | 5 | 10.06 | -1.74 |
|  | SpA | 7 | 7.51 | -0.20 | 5 | 6.32 | -0.56 |
|  | Psoriasis | 4 | 4.71 | -0.34 | 9 | 3.96 | 2.65 |
|  | IBD | 5 | 9.42 | -1.55 | 15 | 7.93 | 2.70 |
|  | Other | 6 | 6.45 | -0.19 | 7 | 5.43 | 0.71 |
| Intermediate Symptomatic | Control | 280 | 248.62 | 3.57 | 112 | 156.30 | -6.05 |
|  | RA | 29 | 48.78 | -3.41 | 56 | 30.66 | 5.24 |
|  | SpA | 23 | 30.65 | -1.62 | 25 | 19.27 | 1.46 |
|  | Psoriasis | 14 | 19.21 | -1.37 | 8 | 12.07 | -1.29 |
|  | IBD | 46 | 38.42 | 1.45 | 29 | 24.15 | 1.11 |
|  | Other | 20 | 26.33 | -1.44 | 29 | 16.55 | 3.40 |
| Oligo-/Asymptomatic | Control | 207 | 170.78 | 4.77 | 437 | 464.06 | -2.58 |
|  | RA | 17 | 33.50 | -3.29 | 112 | 91.04 | 3.03 |
|  | SpA | 14 | 21.05 | -1.73 | 68 | 57.20 | 1.92 |
|  | Psoriasis | 13 | 13.19 | -0.06 | 41 | 35.85 | 1.14 |
|  | IBD | 19 | 26.39 | -1.64 | 64 | 71.70 | -1.24 |
|  | Other | 13 | 18.09 | -1.34 | 47 | 49.15 | -0.41 |
| Data are presented as absolute number (n). IBD: inflammatory bowel disease, IMIDs: immune mediated inflammatory diseases, RA: rheumatoid arthritis, SpA: spondyloarthritis. | | | | | | | |

| **Supp. Table 3: The distribution of participants according to treatment groups by frequency clusters** | | | | | | | |
| --- | --- | --- | --- | --- | --- | --- | --- |
| Symptom frequency clusters | Subclusters of symptom frequency | | | csDMARD | bDMARD | tsDMARD | Glucocorticoid |
| Polysymptomatic | | Higher  n (%) | 6 (5.9) | | 20 (19.2) | 0 (0.0) | 3 (3.0) |
|  |  | Lower  n (%) | 9 (10.6) | | 21 (24.7) | 1 (1.2) | 10 (11.8) |
| Intermediate Symptomatic | | Higher  n (%) | 25 (6.1) | | 96 (23.3) | 4 (1.0) | 21 (5.1) |
|  |  | Lower  n (%) | 58 (22.4) | | 69 (26.6) | 0 (0.0) | 36 (13.9) |
| Oligo-/Asymptomatic | | Higher  n (%) | 23 (8.1) | | 48 (17.0) | 1 (0.4) | 18 (6.4) |
|  |  | Lower  n (%) | 96 (12.5) | | 184 (23.9) | 11 (1.4) | 53 (6.9) |
| Data are presented as absolute number (n) with proportion (%).bDMARDs: biologic disease-modifying anti-rheumatic drugs, csDMARDs: conventional synthetic disease-modifying anti-rheumatic drugs, tsDMARDs: target synthetic disease-modifying anti-rheumatic drugs. | | | | | | | |

**Supplementary Figure 1-legend:**

Standardized residuals by diagnosis (through analysing systemic autoimmune diseases, i.e. systemic lupus erythematosus, autoimmune inflammatory myopathies and systemic sclerosis in a common category)

**Supplementary Figure 2-legend:**

Standardized residuals that summarize the difference between observed and expected number of individuals in each cluster. Values lower than <-2 or higher than >2 were interpreted as an important deviation from the expected counts.

Figure 2A: Standardized residuals by csDMARDs, b-tsDMARDS

Figure 2B: Standardized residuals by glucocorticoid treatment: Standardized residuals with medication subgroups showed no association between symptom clusters.

**Supplementary Figure 3-legend:**

Standardized residuals by treatment subgroups
