# Supplementary material for: Paucity of viral infection symptoms in patients with immune-mediated inflammatory diseases
Source: BMJ Open. 2025 Jan 7;15(1):e088486. doi: 10.1136/bmjopen-2024-088486 (PMC11749532; doi:10.1136/bmjopen-2024-088486)
Supplement: online supplemental file 7 [file bmjopen-15-1-s007.pdf]

M3 SARS-CoV-2 Follow Up

Datum: ______________

Questionnaire

Universitätsklinikum Erlangen

LAURIS-Etikett

mit **Patienten-Nr.**

falls vorhanden

Nachname: ____________________ Vorname: __________________

Geburtsdatum: ________________

Größe (cm): ___________________ Gewicht (kg): ________________

Raucher: ☐ Ja ☐ Früher ☐ Nein

Name der Immunerkrankung (z.B. Rheumatoide Arthritis): ___________________________ ☐ Keine

Art der Immuntherapie (z.B. Adalimumab): ___________________________ ☐ Keine

Einnahme Glukokortikoide (z.B. Cortison, Prednisolon): ☐ Ja ☐ Nein

---

- Leiden Sie **derzeit** an **einer** oder an **mehreren** der hier aufgeführten Erkrankungen?
  - ☐ Diabetes
  - ☐ Pollenallergie
  - ☐ Thrombose
  - ☐ Darmentzündung
  - ☐ Bluthochdruck
  - ☐ Rheuma
  - ☐ Schuppenflechte
  - ☐ Chronische Lungenerkrankung (z.B. Asthma, COPD)
  - ☐ Keine der hier aufgeführten Erkrankungen
- Wurden bei Ihnen bereits **in der Vergangenheit eine** oder **mehrere** der nachfolgend aufgeführten Erkrankungen festgestellt?
  - ☐ Herzinfarkt
  - ☐ Angina pectoris
  - ☐ Krebserkrankung
  - ☐ Keine

- Hatten Sie **direkten Kontakt** zu einem **bestätigten** Verdachtsfall mit SARS-CoV-2?

  ☐ Nein ☐ Ja

  Falls ja, handelte es sich dabei um einen Kontakt innerhalb des eigenen Haushalts (z.B. Partner, Kinder)? ☐ Nein ☐ Ja

- Wurden Sie bereits mittels **Rachenabstrich** (**PCR-Test**) auf eine potentielle SARS-CoV-2-Infektion getestet, weil Sie eine mögliche Infektion vermutet hatten? Hiervon ausgenommen sind symptomlose Tests (z.B. Schnelltests in Apotheken)

  ☐ Nein ☐ Ja

  ➢ Falls ja, wurden Sie jemals mittels PCR-Testung **positiv** auf das SARS-CoV-2-Virus getestet?

  ☐ Nein ☐ Ja – Datum des positiven PCR-Tests: ____________ (falls unbekannt, ungefähre Angabe)

  ➢ Hatten Sie im zeitlichen Zusammenhang während Ihres positiven PCR-Tests Symptome, die auf eine COVID-Erkrankung hindeuten.

  ☐ Ja, ich hatte Symptome ☐ Nein, ich hatte keine Symptome

- Wann wurden Sie zuletzt gegen die Grippe (**Influenza**) geimpft?

  ☐ Noch nie ☐ Zuletzt an folgendem Datum: ____________________ (falls unbekannt, ungefähre Angabe)

- Hatten Sie **eines** oder **mehrere** der nachfolgend aufgeführten Symptome in den vergangenen **4 Wochen**?

☐ Fieber	☐ Reizhusten	☐ Schnupfen	☐ Halsschmerzen
☐ Durchfall	☐ Gliederschmerzen	☐ Kopfschmerzen	☐ Kurzatmigkeit
☐ Geruchsverlust	☐ Nachtschweiß	☐ Neu-aufgetretene Erschöpfung	

☐ Keine der hier aufgeführten Symptome

Bitte wenden!

Datum: ______________

COVID-19 (Corona) Impfung

An welchem Datum wurden Sie gegen SARS-CoV-2 geimpft? Geben Sie bitte bei **jedem Datum** die Art des Impfstoffs an, insbesondere bei einer Impfung mit **zwei unterschiedlichen Präparaten**:

Datum **1. Impfung**: ____________	Datum **2. Impfung**: ____________	Datum **3. Impfung**: ____________
☐ BioNTech/Pfizer, Comirnaty	☐ BioNTech/Pfizer, Comirnaty	☐ BioNTech/Pfizer, Comirnaty
☐ Moderna COVID-19 Vaccine	☐ Moderna COVID-19 Vaccine	☐ Moderna COVID-19 Vaccine
☐ AstraZeneca, Vaxzevria	☐ AstraZeneca, Vaxzevria	☐ AstraZeneca, Vaxzevria
☐ Johnson & Johnson / Janssen	☐ Johnson & Johnson / Janssen	☐ Johnson & Johnson / Janssen
☐ Anderer:	☐ Anderer:	☐ Anderer:
____________________________	____________________________	____________________________

- Wurden Sie darüber hinaus **weitere Male** gegen SARS-CoV-2 geimpft? ☐ Nein ☐ Ja

  Falls ja, an welchem Datum und mit welchem Impfstoff
  - Datum: ________________ Impfstoff: ____
  - Datum: ________________ Impfstoff: ____

- Welche der nachfolgend aufgeführten Symptome hatten Sie innerhalb von **7 Tagen** nach **der jüngsten** bzw. Ihrer **zuletzt erfolgten Impfung** gegen SARS-CoV-2.

  - **Lokale Reaktionen / Symptome**

    ☐ Schmerzen an der Injektionsstelle ☐ Rötung

    ☐ Schwellung
  - **Systemische Reaktionen / Symptome**

    ☐ Müdigkeit ☐ Kopfschmerzen

    ☐ Gelenkschmerzen ☐ Muskelschmerzen

    ☐ Schüttelfrost ☐ Fieber (≥ 38 °C)

    ☐ Übelkeit / Erbrechen ☐ Durchfall

    ☐ Lymphknotenschwellung ☐ Neurologische Beschwerden (z.B. Gesichtslähmungen)

    ☐ Andere: ____

  ☐ Keine der hier aufgeführten Symptome

- Wurde bei Ihnen **nach** erfolgter Impfung eine **neue** Erkrankung diagnostiziert?

  ☐ Nein ☐ Ja Falls ja, welche Erkrankung(en) wurden bei Ihnen neu diagnostiziert?

- Hat sich eine Ihrer **vorne angegebenen** Erkrankung **nach** erfolgter Impfung insgesamt **verschlechtert**?

  ☐ Nein ☐ Ja Wenn ja, welche Ihrer Erkrankungen und inwiefern hat sich diese verschlechtert?

  ☐ Ich musste wegen einer Verschlimmerung der Erkrankung einen Arzt aufsuchen

  ☐ Mir wurde ein neues Medikament verschrieben: ________________________

  ☐ Ich musste die Medikamentendosis aufgrund der Verschlechterung ändern

  ☐ Ich musste ins Krankenhaus eingeliefert werden oder mein Aufenthalt wurde verlängert

  ☐ Ich habe mich einer Operation oder einer Bildgebung (CT/MRT) unterzogen

Vielen Dank!
