# Supplementary material for: Paucity of viral infection symptoms in patients with immune-mediated inflammatory diseases
Source: BMJ Open. 2025 Jan 7;15(1):e088486. doi: 10.1136/bmjopen-2024-088486 (PMC11749532; doi:10.1136/bmjopen-2024-088486)
Supplement: Supplementary file 8 [file bmjopen-2024-088486.reviewer_comments.pdf]

PEER REVIEW HISTORY

BMJ Open publishes all reviews undertaken for accepted manuscripts. Reviewers are asked to complete a checklist review form and are provided with free text boxes to elaborate on their assessment. These free text comments are reproduced below.

ARTICLE DETAILS

Title (Provisional)

Paucity of viral infection symptoms in patients with immune-mediated inflammatory diseases

Authors

Yalcin Mutlu, Melek; Kleyer, Arnd; Kroenke, Gerhard; Fagni, Filippo; Temiz, Selahattin Alp; Meder, Christine; Dietrich, Peter; Orlemann, Till; Mößner, Johanna; Schoenau, Verena; Bohr, Daniela; Schuster, Louis; Hartmann, Fabian; Minopoulou, Ioanna; Leppkes, Moritz; Ramming, Andreas; Pachowsky, Milena L.; Schuch, Florian; Ronneberger, Monika; Kleinert, Stefan; Hueber, Axel J.; Manger, Karin; Manger, Bernhard; Atreya, Raja; Berking, Carola; Sticherling, Michael; Neurath, Markus F; Schett, Georg; Simon, David; TASCILAR, Koray

---

VERSION 1 - REVIEW

**Reviewer**	**1**
**Name**	**Rob , Filip**
**Affiliation**	**Charles University, Dermatovenerology**
**Date**	**30-May-2024**
**COI**	**None**

The manuscript deals with the important issue of viral infections in patients with immune-related diseases and the effect of targeted therapy. The text is written clearly and comprehensibly, the methodology is explained in detail, the results are clear, including tables and graphs. The conclusions of the discussion correspond to the results. The limitations of the work are sufficiently mentioned.

**Reviewer**	**2**
**Name**	**Mavragani, CP**
**Affiliation**	**National and Kapodistrian University of Athens, Department of Physiology**
**Date**	**05-Jun-2024**

COI **None**

In the present report the authors report a lower frequency of viral infection symptoms in IMID patients compared to controls. This data is novel and might imply the intrinsic or treatment induced mobilization of protective immune responses against viral antigens in these patients. However, several issues arise:

1.It is not clear how Poisson regression analysis was performed. Age and sex associations were detected for both IMID patients and controls? It would be desirable to display associations with viral symptoms separately for IMID and controls.

2. Another potential concern if that the other IMID group is greatly heterogeneous including both systemic autoimmune and inflammatory patients. Given the prominent upregulation of type I IFNs in systemic lupus erythematosus, Sjogren's syndrome, systemic sclerosis and dermatomyositis, it would be much more informative to report the prevalence of viral symptoms in this group separately.

3.Moreover, previous studies in inflammatory myositis, Sjogren's syndrome and psoriasis revealed augmentation of type I IFN pathways following anti-TNF treatment. Therefore it would be also interesting to display the prevalence of viral symptoms according to the type of biological treatment (anti-TNF vs non anti-TNF) and add these concepts in the discussion section.

4. In the same context, it would be interesting to report old data suggesting that systemic lupus erythematosus on remission displayed increased prevalence of herpes zoster infections presumably due to suppression of endogenous type I IFN responses following treatment.

VERSION 1 - AUTHOR RESPONSE

Reviewer: 1
Dr. Filip Rob, Charles University
Comments to the Author:
The manuscript deals with the important issue of viral infections in patients with immune-related diseases and the effect of targeted therapy. The text is written clearly and comprehensibly, the methodology is explained in detail, the results are clear, including tables and graphs. The conclusions of the discussion correspond to the results. The limitations of the work are sufficiently mentioned.

-We thank the reviewer for the positive feedback.

Reviewer: 2
Dr. CP Mavragani, National and Kapodistrian University of Athens
Comments to the Author:
In the present report the authors report a lower frequency of viral infection symptoms

in IMID patients compared to controls. This data is novel and might imply the intrinsic or treatment induced mobilization of protective immune responses against viral antigens in these patients. However, several issues arise:

1.It is not clear how Poisson regression analysis was performed. Age and sex associations were detected for both IMID patients and controls? It would be desirable to display associations with viral symptoms separately for IMID and controls.

-The model summary including exponentiated model coefficients were already reported in Table-3, there was no additional detail to the regression analysis. Age and sex associations were detected across the overall population regardless of IMID status which is a well-known phenomenon, i.e. viral infection symptoms are less likely to be manifested with increasing age and in males compared to females. Our aim in including age and sex in the analyses did not necessarily aim to reproduce or dispute this pattern but was principally for adjustment, since IMID patients were older and were more likely to be females. This point is now clarified in the discussion section. We did not understand very well how the associations with viral symptoms would separately be reported for IMIDs and controls, since association implies a relative or an absolute comparison, i.e. in IMID patients versus controls. We have already tabulated the numbers and proportions of individuals showing symptoms overall and in subgroups in table 2 and also in supplementary tables as observed and expected numbers for each cluster and each group as well.

2. Another potential concern if that the other IMID group is greatly heterogeneous including both systemic autoimmune and inflammatory patients. Given the prominent upregulation of type I IFNs in systemic lupus erythematosus, Sjogren's syndrome, systemic sclerosis and dermatomyositis, it would be much more informative to report the prevalence of viral symptoms in this group separately.

-Other IMID group includes IMID diagnoses for which there were less than 50 patients each. As the reviewer would also appreciate, these individual diagnoses would not have a substantial "weight" in the IMID population or would not alter our conclusions on what has been observed in the overall analyses. In addition, upregulation of type I IFNs is not unique to these conditions and also observed in RA and psoriasis which constitute a substantial proportion of our IMID group as we have mentioned in our discussion. There was a total of 42 patients with the diagnoses mentioned by the reviewer, we have nevertheless repeated the residual analyses and regression by separately categorizing these patients and did not observe any particular deviation from expected numbers as added now to the supplement.

3.Moreover, previous studies in inflammatory myositis, Sjogren's syndrome and psoriasis revealed augmentation of type I IFN pathways following anti-TNF treatment. Therefore, it would be also interesting to display the prevalence of viral symptoms according to the type of biological treatment (anti-TNF vs non-anti-TNF) and add these concepts in the discussion section.

-We have conducted additional analyses separating TNFs and other targeted treatments, but we did not specifically see an overrepresentation or underrepresentation of TNF users in any symptom cluster. These seem to follow the diagnoses rather than the specific treatments. These analyses are also added to the supplement.

4. In the same context, it would be interesting to report old data suggesting that systemic lupus erythematosus on remission displayed increased prevalence of herpes zoster infections presumably due to suppression of endogenous type I IFN responses following treatment.

-We thank the reviewer for this comment. We mentioned increased incidence of herpes zoster infections in SLE patients with lower disease activity under immune suppressive therapy. Moreover, we also added a new paragraph focusing on potential defective IFN responses in IMID patients. Additionally we have expanded the discussion on IFN responses to give further perspective and comment on the relevance to our findings.

VERSION 2 - REVIEW

**Reviewer**	**2**
**Name**	**Mavragani, CP**
**Affiliation**	**National and Kapodistrian University of Athens, Department of Physiology**
**Date**	**30-Aug-2024**
**COI**	**None**

No further comments.

VERSION 2 - AUTHOR RESPONSE

Dear Dr. Philips,

In line with your suggestions these corrections were made:

1- The abstract is formatted according to journal instructions.

2- Sections that did not comply with the journal format were removed.

3- Selected versions of the questionnaire utilized throughout the study were added as "Supplemental Information files".

Best regards,

Dr. Koray Tascilar

**** Response to Reviewer - Author's Response.docx ****
